# The evaluation of renewable energy alternatives in Turkey using intuitionistic-fuzzy EDAS methodology

**DOI:** 10.1007/s11356-023-31816-7

**Published:** 2024-02-01

**Authors:** Cüneyt Dumrul, Faik Bilgili, Fulya Zarali, Yasemin Dumrul, Zerrin Kiliçarslan

**Affiliations:** 1 Faculty of Economics and Administrative Sciences, Erciyes University, Kayseri, Turkey; 2 Kayseri University Faculty of Engineering, Architecture and Design Department of Industrial Engineering, Kayseri, Turkey

**Keywords:** Multi-criteria decision-making problems, Intuitionistic Fuzzy-EDAS, Renewable energy, P18, Q20, Q42, D81

## Abstract

In recent years, high energy costs, increases in carbon emissions, and concerns about energy supply security have led countries to prioritize renewable energy sources in their sustainable energy policies. The selection and ranking of alternative renewable energy sources is a critical issue in establishing an effective energy policy and ensuring environmental improvement at the national and global levels. This study evaluates Turkey’s best renewable energy options using the institutional fuzzy assessment based on distance to mean solution (IF-EDAS) method and aims to find Turkey’s best renewable energy alternative. The decision model alternatively uses wind, solar, geothermal, biomass, wave, hydroelectric, and hydrogen energy options. According to the study’s empirical findings, while the best alternative renewable energy source for Turkey is solar energy, the best criterion in terms of criteria evaluation is “Technology Maturity”. The study also carried out sensitivity analysis, and the results were shared.

## Introduction

Priorities in energy policy are similar to stages in Maslow’s pyramid of human needs. Maslow’s pyramid of human needs is based on historical evidence. These stages are access to energy, security of supply, energy costs, environmental issues, and social compliance. This ranking shows that ecological and social priorities are at the bottom of traditional energy policies. Indeed, historical national energy policy observations reflect countries’ efforts to obtain commercial energy in the first stage. The next stage is the security of supply, followed by cost-effectiveness. In the late 1970s, industrialized countries started considering the efficiency of natural resources. Social acceptability has followed this stage since the late 1980s. The last three stages reflect the foundations of sustainable development (Frei 2004).

The intensive use of fossil fuels with the 1st Industrial Revolution has caused today’s climate change, global warming, and environmental degradation to reach a level that greatly threatens living life in the future. In addition to fossil fuels, excessive demand for natural resources in economic activity puts pressure on the ecosystem. This pressure manifests in increasing air and ocean temperatures, decreasing agricultural production, melting glaciers, rising sea levels, unpredictable precipitation, extinction of wildlife, the emergence of new viruses, and the inability to control pandemics. The mentioned problems have led to the two most talked about concepts in the “sustainability” literature, especially for developing countries, being “economic sustainability” and “environmental sustainability.” Many developing economies are still dependent on fossil fuels, particularly coal and oil, requiring greater investment in renewable energy sources to achieve net zero emissions globally. Increasing these investments requires the adoption of taxation and the determined implementation of taxation and subsidy policies that will encourage the reduction of carbon emissions (Balsalobre-Lorente et al. 2023; Do et al. 2022; Jahanger et al. 2022; Rafei et al. 2022).

In Turkey, whose industrialization started relatively late compared to Western economies, priorities in energy policies have been different and late compared to developed Western economies. While there were rapid industrialization strategies and traditional energy policies in the 1980s without considering environmental priorities, after the 2001 financial crisis, the Turkish economy, which is one of the largest countries in Europe in terms of energy balance, showed a relatively rapid and stable economic growth compared to previous years. In parallel with the increasing energy production accompanying its economic growth, the share of fossil energy sources in energy production is high. In addition to the adverse environmental effects of fossil resources, the Turkish economy is dependent on imports of these resources. These two problems increase long-term energy usage costs in the Turkish economy. As a result, Turkey has changed its energy supply choices to more environmental and social priorities in recent decades.

A fundamental problem for Turkey regarding energy balances is the high share of fossil energy resources in the total energy production supply. Fossil fuels are a disadvantage in sustainable development for Turkey as they increase foreign dependency on energy and carbon emissions. In the world energy market, where fossil fuels are scarce, Turkey’s dependence on fossil fuels causes economic, political, and strategic problems. On the other hand, although Turkey has a higher potential for renewable energy resources than fossil energy resources, the share of renewable energy resources in Turkey’s energy consumption in 2019 is only 10% (IEA 2021b; Kaya and Kahraman 2010; MENR 2022). According to IEA (2021b), renewable energy sources meet approximately 10% of Turkey’s total final energy consumption. However, the primary energy production of non-renewable and renewable resources is insufficient to meet the country’s demand. In addition, Turkey imports more than 70% of the energy resources required to meet energy consumption. All the problems mentioned show that renewable energy and the right choice among alternatives are vital for Turkey to ensure sustainable growth.

Within the above framework, this study aims to determine the most optimal ranking of Turkey’s renewable energy production preferences, reducing foreign dependency on energy and environmental problems arising from energy production and consumption. In addition, to establish an effective energy policy, choosing an appropriate renewable energy source is important to develop new economic markets, create employment opportunities, and improve the energy use structure (Zhang et al. 2015). This study followed the EDAS approach, which was proposed as a new inventory MCDM method (Keshavarz et al. 2015). The method is based on determining the mean solution value and obtaining an evaluation score based on positive and negative deviations from this value. This method determines the best solution by calculating the distance to the average solution, thus providing a high-efficiency solution (Ecer 2020). In this study, the EDAS method is modeled using intuitionistic fuzzy numbers to overcome uncertainty. The advantages mentioned are the reason for the preference for this method.

In summary, the optimal allocation of energy resources is crucial for Turkey, which has limited financial resources and has experienced many crises since the 1980s. Turkey uses fossil energy resources proportionately more in total production, and this causes environmental negativities. In addition, Turkey is largely dependent on foreign sources of fossil fuels, which is an important reason for the country's current account deficit problem. For Turkey, which has a high renewable and clean energy potential, which renewable energy option will be preferred according to which criteria is a complex optimization and economic policy problem. This is the main motivation of this study. MCDM is one of the best options for solving a complex optimization problem with many criteria and options. This study uses the Intuitionistic-Fuzzy EDAS methodology, one of the most advanced and up-to-date versions of the MCDM methods, for the first time in this field and also provides the opportunity to generalize it for countries with similar problems.

This study contributed to the literature on the proposed renewable energy source selection method, and IF sets. Although many studies use the classical EDAS method, there are few studies with the EDAS method integrated with the intuitionistic fuzzy set. The originality of this study is the first use of new criteria, consisting of two main and seven sub-criteria, which have not been used in the literature before, in evaluating alternative renewable energy sources with IF-EDAS analysis. The rest of the paper is organized as follows: the second chapter briefly overviews Turkey’s energy balances and renewable energy policies. While the third section gives theoretical information about the IF-EDAS methodology, the fourth and final section includes the application. The last section presents the results of the study and policy recommendations.

## Turkey's energy balances and renewable energy policies: a brief overview

In Turkey, a developing economy, energy consumption has increased rapidly, especially in the last two decades, with urbanization, increasing demographic trends, economic growth, and rising per capita income (Bulut and Muratoglu 2018). Turkey’s total final energy consumption was 37.714 KTOE in 1990, reaching 101.143 KTOE in 2020. In response to Turkey’s rapidly increasing energy demand, Turkey’s energy supply is also growing. Turkey’s total energy supply (TES) was 51.605 KTOE in 1990 and 146.134 KTOE in 2020 (EUROSTAT 2022).

The rapidly increasing population (annual average growth rate is roughly 1.3%) with the increasing external migration in recent years, the industry’s continuous growth since the 1980s, and the high average economic growth (about 5% per year) also affect the energy balances in the Turkish economy. Although Turkey’s energy consumption has increased significantly due to rapid population growth, urbanization, widespread industrialization, and income, imports mostly meet energy production. This situation increases Turkey’s current account deficit and external borrowing to finance the current account deficit. Turkey’s dependence on foreign energy resources has increased dramatically, especially since early 1990, and has run around 70% since the early 2000s (Bulut and Muratoglu 2018; Gönül et al. 2021; Kok and Benli 2017).

Turkey’s rapid economic and population growth has increased its energy demand in the last two decades and caused many critical problems. The first and perhaps the most important of these problems is import dependency on energy. Turkey heavily depends on oil and natural gas imports, as only about a quarter of its energy demand is met by domestic production (Sharif et al. 2020). Therefore, one of the crucial reasons for the current account deficit, an economically significant problem in Turkey, is the dependence on imports in energy resources (Bulut and Muratoglu 2018). Moreover, as domestic non-renewable energy resources are limited and inadequate, energy imports take the highest share of Turkey’s total imports. Turkey’s energy production is insufficient to meet the country’s ever-increasing energy demand, causing approximately 75% of its total energy to be completed by imports (Sharif et al. 2020). Therefore, Turkey is a net energy importer due to the shortage of energy supply and increasing energy consumption, and this import enlarges the foreign trade deficit (Bulut and Muratoglu 2018). Turkey’s dependence on energy imports and fluctuations in energy prices cause energy supply risk, make the economy more vulnerable to external shocks, and raise concerns about the sustainability of economic development. For these reasons, energy supply security remains one of Turkey’s primary energy policy priorities.

The second significant problem with Turkey’s energy is environmental pollution. The high use of fossil energy resources increases carbon and other harmful emissions. In 2018, Turkey’s share of fossil energy resources was approximately 86% (natural gas 28%, oil 29%, and coal 29%). According to the IEA (2021a), total ${{\text{CO}}}_{2}$ emissions, which is one of the sustainable development indicators, increased from 128.76 (mt of ${{\text{CO}}}_{2}$) in 1990 to 366.11 (mt of ${{\text{CO}}}_{2}$) in 2020 in Turkey. On the other hand, the share of energy obtained from renewable sources is only 14% in 2019, which is very low for sustainable development. The percentage of renewable energy types in TPES is 4% hydro, 2% biofuels and waste, 8% wind, solar, etc. (IEA 2021b; Sharif et al. 2020). In the literature, there is a remarkable consensus on reducing the use of fossil fuels in Turkey for various reasons (concerns about the depletion of fossil fuels, effects on climate change, volatility of prices, etc.) (Bulut and Muratoglu 2018). While the potential of non-renewable energy resources such as petroleum and lignite coal to meet the energy needs of the country is low in terms of reserves in Turkey, renewable energy sources such as solar, water, wind, geothermal, and biomass have an important potential to generate energy in a way that supports sustainable development. However, the high level of energy consumption causes the energy produced by renewable energy sources to be insufficient, and therefore, the use of non-renewable energy sources increases continuously (IEA 2021b; Önder 2021).

In particular, Turkey’s high dependence on fossil fuel imports has led it to prioritize energy supply security as one of the fundamentals of its energy strategy. To reduce these problems, policy-makers have implemented policies to restructure Turkey’s energy system to rationalize the growth in energy demand in Turkey, lower energy prices for consumers, slow down the increase in energy imports, and reduce environmental problems caused by energy consumption. For these problems, policy-makers have implemented policies to restructure Turkey’s energy system to rationalize the growth in energy demand in Turkey, lower energy prices for consumers, slow down the increase in energy imports, and reduce environmental problems caused by energy consumption. In particular, Turkey’s high dependence on fossil fuel imports has led it to prioritize energy supply security as one of the fundamentals of its energy strategy. In addition to these policies, policies including increasing domestic oil and natural gas exploration activities and production, diversifying oil and natural gas supply sources and related infrastructure, increasing renewable energy production, and increasing energy efficiency are implemented. In Turkey, considerable diversification has been observed in the energy composition over the past decade. Turkey’s first nuclear power plant will be operational in 2023, leading to more diversification of the country’s design in energy resources (Bilgili et al. 2022; IEA 2021b).

## Empirical literature

In the empirical literature, studies use different numbers and types of main criteria, sub-criteria, and multi-criteria decision-making methods for selecting the best renewable energy source among various alternatives. Table 1 summarizes the studies that applied fuzzy-based multi-criteria decision-making in evaluating renewable energy sources. Table 1 presents 22 studies using MCDM methods by country analyzed, methodology, alternatives, criteria, and result (best alternative). The ten studies in the table are related to Turkey; two concluded that solar energy, four studies hydro energy, and four studies wind energy is the best renewable energy source. In general, studies in the empirical literature use economic, technical, social, and environmental criteria. In addition to these criteria in the literature, some studies use political criteria (Bilgili et al. 2022; Saraswat and Digalwar 2021; Solangi et al. 2020; Wang et al. 2021) or social and political criteria in the same classification as sociopolitical criteria (Al Garni et al. 2016; Çolak and Kaya 2017; Ertay et al. 2013).

**Table 1:** Summary of a literature review using MCDM to evaluate the selection of renewable energy

Author(s)	Country	Methodology	Alternatives		Criteria (sub-criteria)	Result (Best Alternative)
Bilgili et al. (2022)	Turkey	- IF-TOPSIS	- Wind - Solar - Geothermal - Hydraulics	- Biomass - Wave - Hydrogen	- Economic (6) - Technical (6) - Social (4) - Environmental (5) - Political (4)	Solar
Saraswat and Digalwar (2021)	India	- Shannon’s entropy MCDM	- Thermal - Gas - Nuclear - Solar	- Wind - Biomass - Hydro	- Economic (7) - Technical (5) - Social (4) - Environmental (4) - Political (4) - Flexibility (2)	Solar
Karatop et al. (2021)	Turkey	- Fuzzy AHP-EDAS - Fuzzy FMEA	- Hydropower - Wind - Solar	- Geothermal - Biomass	- Cost (3) - Political (3) - Technology (3) - Environmental (9) - Construction-Management (11)	Hydropower
Wang et al. (2021)	Vietnam	- G-AHP - WASPAS	- Wind - Solar	- Solid waste - Biomass	- Economic (3) - Technical (5) - Environmental (3) - Social (3) - Political (2)	Solar
Li et al. (2020)	China	- ANP - WSM - TOPSIS - VIKOR - PROMETHE - ELECTRE VIKOR	- Solar - Wind - Hydro	- Geothermal - Biomass	- Energy (4) - Economic (5) - Technical (2) - Environment and carbon (6) - Social (3)	Hydro
Deveci et al. (2020)	Turkey	- IVIF - IVIF CODAS	- Hydropower - Wind-onshore - Solar PV	- Geothermal - Biomass power	- Technological (7) - Environmental (3) - Socio-political (4) - Economic (3)	Wind-onshore
Yazdani et al. (2020)	Saudi Arabia	- EDAS - VIKOR - MABAC	- Solar PV - Solar thermal - Wind	- Geothermal - Biomass	- Economic (3) - Technical (2) - Social (1) - Environmental (2)	Wind
Solangi et al. (2020)	Turkey	- Fuzzy WASPAS	- Solar - Wind - Hydro	- Geothermal - Bioenergy	- Environmental (3) - Economic (4) - Technical (4) - Politic (3) - Social (3)	Wind
Yilan et al. (2020)	Turkey	- MCDA with WSM	- Natural gas - Coal - Hydropower with dam - Hydropower	- Run-of-river type - Wind-onshore - Geothermal - Solar PV	- Economic (1) - Technical (4) - Environmental (3) - Socio-economic (4)	Hydropower with the dam
Karakaş and Yildiran (2019)	Turkey	- Modified Fuzzy AHP	- Hydro - Wind - Solar	- Biomass - Geothermal	- Technical (2) - Economic (2) - Environmental (2) - Social (2)	Solar
Lee and Chang (2018)	Taiwan	- WSM - VIKOR - TOPSIS - ELECTRE	- Hydro - Wind power - Solar energy	- Biomass - Geothermal	- Economic (3) - Technical (3) - Environmental (2) - Social (2)	Hydro
Çolak and Kaya (2017)	Turkey	-Hesitant fuzzy TOPSIS	- Solar - Wind - Hydro - Geothermal	- Biomass - Hydrogen - Wave	- Energy Source Quality (3) - The technique (5) - Environment (5) - Technological (6) - Economic (6) - Sociopolitical (4)	Wind
Özkale et al. (2017)	Turkey	- PROMETHE	- Wind - Hydro - Solar	- Biomass - Geothermal	- Economic (4) - Sociological (2) - Environmental (3) - Technical (3)	Hydro
al Garni et al. (2016)	Saudi Arabia	- AHP	- Solar PV - Solar thermal - Wind energy	- Biomass - Geothermal	- Socio-political (7) - Technical (11) - Economic (12) - Environmental (6)	Solar PV
Klein and Whalley (2015)	USA	- Fuzzy TOPSIS	- Natural gas - Nuclear - Hydropower - Wind-onshore - Wind-offshore - Biopower - PV - Coal	- Geothermal-flash - Geothermal-binary - CSP-MB - CSP-FF - CSP-TES	- Economic (1) - Environmental (4) - Social (2) - Technical (1)	Bio-power
Şengül et al. (2015)	Turkey	- Fuzzy TOPSIS	- Regulator - Wind Power Station - Geothermal	- Geothermal Power Station - Hydro Power Station	- Technical (3) - Economic (3) - Environmental (2) - Social (1)	Hydro power station
Abdullah and Najib (2014)	Malaysia	- Intuitionistic Fuzzy AHP	- Conventional - Nuclear - Solar - Wind	- Hydraulic - Biomass - CHP	- Technical (2) - Economic (2) - Environmental (3) - Social (2)	Nuclear
Ahmad and Tahar (2014)	Malaysia	- AHP	- Solar PV - Hydropower	- Biomass - Wind	- Technical (3) - Economic (4) - Social (2) - Environmental (3)	Solar PV
Stein (2013)	Malaysia	- AHP	- Solar PV - Wind - Hydro - Geothermal - Biomass	- Nuclear - Coal - Oil - Gas	- Financial (5) - Technical (3) - Environmental (2) - Social/Economic/Political (3)	Wind
Ertay et al. (2013)	Turkey	- MACBETH Fuzzy AHP	- Hydropower - Wind - Solar	- Biomass - Geothermal	- Technological (5) - Environmental (3) - Socio-Political (4) - Economic (3)	Wind
Amer and Daim (2011)	Pakistan	- AHP	- Wind - Solar PV	- Solar thermal - Biomass	- Technical (7) - Economic (5) - Social (3) - Environmental (3) - Political (2)	Biomass
Nigim et al. (2004)	Canada	- AHP - SIMUS	- Solar PV - Solar thermal - Wind	- Geothermal - Micro-hydro	- Resource availability (1) - Ecological impact (2) - Technical feasibility (5) - Financial viability (4) - Educational potential (3) - Socio-economic benefits (2)	Solar

Numbers in parentheses indicate sub-criteria numbers

As seen in Table 1, although many studies use the classical EDAS method, few use the EDAS method integrated with the intuitionistic fuzzy set. This study contributes to the literature by employing the IF-EDAS method for the first time in selecting renewable energy alternatives and criteria. Besides, considering new criteria for renewable energy selection is another contribution of this study to the literature. Table 1 also includes ten studies on Turkey using MCDM methods. Of these ten studies on renewable energy rankings, four recommend hydro, four recommend wind, and two recommend solar energy.

## Methodology

A number of decision problems (DPs) have been resolved using EDAS, a relatively new approach in the literature, by combining hybrid and system theories with other strategies. It is also becoming a more and more common MCDM approach. The method depends on determining the distance to the average solution, which entails producing an assessment score based on positive and negative departures from the DP’s average solution value (Yildirim and Meydan 2021).

Studies using the expanded EDAS method using IF numbers (IFN) are uncommon despite the fact that the EDAS approach is frequently employed in research. A combined IF-EDAS approach with an IF cluster is suggested for this purpose. To this end, the IF-EDAS approach in conjunction with the IF set is recommended to assess Turkey's renewable energy options.

### EDAS

Keshavarz et al. (2015) initially introduced the EDAS methodology as a novel MCDM method for inventory classification DP in the literature. It calculates the average values of the criteria rather than relying on the gap between ideal and non-ideal values, as in earlier distance-based methods. Alternatives are evaluated using this mean value combined with positive and negative distance metrics (Yildirim and Meydan 2021). The first two measurements in the EDAS technique are the positive distance from average (PDA) and the negative distance from average (NDA). These measurements can show the differences between each alternate choice and the average response. As a result, the ideal solution is indicated by greater PDA and lower NDA values (Kahraman, et al. 2017; Ecer 2020).

The following is a description of the method’s steps.

- Step 1: Firstly, criteria and alternatives are determined.
- Step 2: If ${x}_{ij}$ is the performance rating of the *i*th alternative $(A=\left\{{A}_{1},{A}_{2},\dots \dots {A}_{n}\right\}$), in accordance with the in terms of the *j*th criterion $(C=\left\{{C}_{1},{C}_{2},\dots \dots {C}_{m}\right\}$). As a result, when creating the interval decision matrix X and choosing the importance of each criterion, the following table and factors should be considered:
  1 $$X={\left[{x}_{ij}\right]}_{m\times n}$$
  2 $$W={\left[{w}_{j}\right]}_{1\times m}$$
- Step 3: After the decision matrix is created according to the steps of the EDAS technique, the average solution values for the criteria are determined. The average solution value is obtained by Eq. (3).
  3 $${{\text{AV}}}_{J}=\frac{\sum_{i=1}^{n}{x}_{ij}}{n}$$
- Step 4: Finding the positive distance from the mean solution (PDA) and negative distance from the mean solution (NDA) values for the criteria. PDA and NDA distances are calculated using Eqs. (4–5).
  4 $${{\text{PDA}}}_{\dot{{\text{I}}}J}=\frac{{\text{max}}(0,({x}_{ij}-{AV}_{j})}{{AV}_{j}}$$
  5 $${{\text{NDA}}}_{\dot{{\text{I}}}J}=\frac{{\text{max}}(0,({AV}_{j}-{x}_{ij})}{{AV}_{j}}$$
- Step 5: The weighted total positive distance from the average solution and the negative distance from the average solution ($${SP}_{i},{SN}_{i})$$ are calculated through Eqs. (6–7). The value $${w}_{j}$$ in the equation indicates the importance weight of criterion *j*.
  6 $${{\text{SP}}}_{i}=\sum_{j=1}^{m}{w}_{j}{PDA}_{ij}$$
  7 $${{\text{SN}}}_{i}=\sum_{j=1}^{m}{w}_{j}{NDA}_{ij}$$
- Step 6: Normalization of the weighted total values of the alternatives ($${NSP}_{i},{NSN}_{i})$$. Normalization is calculated using Eqs. (8–9).
  8 $${{\text{NSP}}}_{i}=\frac{{SP}_{i}}{{{\text{Max}}}_{i}({SP}_{i})}$$
  9 $${{\text{NSN}}}_{i}=1-\frac{{SN}_{i}}{{{\text{Max}}}_{i}\left({SN}_{i}\right)}$$
- Step 7: Calculation of assessment scores ${AS}_{i}$. The assessment scores of all alternatives are calculated using Eq. (10). ${AS}_{i}$ takes a value between 0 and 1.
  10 $${AS}_{i}=\frac{1}{2}\left({{\text{NSP}}}_{i}+{{\text{NSN}}}_{i}\right)\mathrm{where }0\le {AS}_{i}\le 1.$$
- Step 8: Ranking of alternatives, the available alternatives are ranked in descending order based on their assessment score. The alternative with the highest evaluation score is considered the best choice.

### IF set

In real-world decision-making situations, verbal-valued criteria are preferred over numerical-valued criteria. Using linguistic variable tools, the weights and levels of importance of the criteria addressed in the problems are established. Fuzzy set theory is a method that permits both verbal judgment and numerical conclusions in situations of ambiguity caused by individual. Fuzzy sets are defined as having a range between [0,1], and the degree of membership is a mathematical representation of fuzzy numbers. By extending Zadeh (1965)’s concept, Atanassov (1986) created the phrase “IF set.” In 1986, Atanassov developed Zadeh’s concept and coined the phrase “IF set.” Atanassov (1986) employed the ideas of the degree of not belonging to the set and hesitation index in IF set theory in addition to Zadeh’s definition. The IF set $A$ in $X$ is expressed as $A=\left\{\left(x,{\mu }_{A}\left(x\right),{\nu }_{A}\left(x\right)\right)|x\in \right\}$ when $X$ is a non-empty set. In IF set theory, it defined the hesitation index as ${\pi }_{A}(x)$, the degree of non-belonging as ${\nu }_{A}\left(x\right)$, and the degree of belonging of the element x to the set $A$ as ${\mu }_{A}\left(x\right)$. The sum of the degrees of belonging and not belonging has a value in the range [0,1] according to IF set theory. 0 ≤ ${\mu }_{A}\left(x\right)+{\nu }_{A}\left(x\right)$  ≤ 1. Whether an element x belongs to set A or not determines the level of hesitation. It is determined by Eq. (11) (Yildirim and Meydan 2021).

11 $${\pi }_{A}\left(x\right)=1-{\mu }_{A}\left(x\right)-{\nu }_{A}\left(x\right)$$

The arithmetic operators for IFN are listed below.

- *Definition 1*: Two IFNs with parameters and a non-zero constant number,$A=({\mu }_{x},{\nu }_{x})$ and $B=({\mu }_{y},{\nu }_{y})$, shall be. These IFN operations are listed below.
  12 $$A\oplus B=({\mu }_{x}+{\mu }_{y}-{\mu }_{x}.{\mu }_{y},{\nu }_{x}.{\nu }_{y})$$
  13 $$A\otimes B=({\mu }_{x}.{\mu }_{y},{\nu }_{x}+{\nu }_{y}-{\nu }_{x}.{\nu }_{y})$$
  14 $$\uplambda .{\text{A}}=\left(1-{\left(1-{\mu }_{x}\right)}^{\uplambda },{{\nu }_{x}}^{\uplambda }\right),\uplambda >0$$
  15 $${A}^{\uplambda }=\left({{\mu }_{x}}^{\uplambda },1-{\left(1-{\nu }_{x}\right)}^{\uplambda }\right),\uplambda >0$$
- *Definition* 2: Let ${\partial }_{x}=({\mu }_{x},{\nu }_{x}))$ be an IFN with parameters.
  16 $$S\left({\partial }_{x}\right)=\left({\mu }_{x}-{\nu }_{x}\right), h\left({\partial }_{x}\right)=\left({\mu }_{x}+{\nu }_{x}\right)$$
  are called as the IFN ${\partial }_{x}$’s scoring function and accuracy function, respectively, where $S\left({\partial }_{x}\right)\in \left[-\mathrm{1,1}\right]$ and $h\left({\partial }_{x}\right)\in \left[\mathrm{0,1}\right]$ represent net membership and accuracy degree, respectively. Later, Xu et al. (2015) changed the score function and defined the new score function in Definition 3.
- Definition 3: Let  ${\partial }_{x}=({\mu }_{x},{\nu }_{x})$ be an IFN. Then
  17 $${S}^{*}\left({\partial }_{x}\right)=\frac{1}{2}\left(S\left({\partial }_{x}\right)+1\right), h\left({\partial }_{x}\right)=\frac{1}{2}\left({\mu }_{x}+{\nu }_{x}\right)$$
  ${S}^{*}\left({\partial }_{x}\right)\in \left[\mathrm{0,1}\right]$ and $h\left({\partial }_{x}\right)\in \left[\mathrm{0,1}\right]$ are obvious.
  Let ${\partial }_{y}=\left({\mu }_{y},{\nu }_{y}\right)$ and ${\partial }_{z}=({\mu }_{z},{\nu }_{z})$ be two IFN with parameters.
  18 $$\mathrm{If }{S}^{*}\left({\partial }_{y}\right)<{S}^{*}\left({\partial }_{z}\right),\mathrm{ then }{\partial }_{y}<{\partial }_{z}$$
  19 $$\mathrm{If }{S}^{*}\left({\partial }_{y}\right)={S}^{*}\left({\partial }_{z}\right),\mathrm{ then }{\partial }_{y}={\partial }_{z}$$

### IF-EDAS method

The decision-makers’ (DM) assessments must be accurate for the decision-making process to be effective. The decision-making process can grow increasingly complicated as the number of criteria and options increases, along with the DM’s level of expertise, perspective, and other factors. The decision-making process contains doubt and ambiguity. We employ IFN to get around this. In this study, the EDAS technique is modeled using IFN. The following steps are launched to model the IF-EDAS approach (Yildirim and Meydan 2021).

- Step 1: The DMs’ weights should be determined. The evaluations made by each member of the decision-making expert group are taken into account to construct the decision matrix. The evaluation of criteria-based alternatives by each expert is aided by the utilizing of language variables. The decision matrix receives the table’s linguistic variables translated to IFN.
  To rate the kth DM, let ${D}_{k}=\left[{\mu }_{k},{\nu }_{k},{\pi }_{k}\right]$ be an IFN. The following Eq. (20) can be followed to compute the weight of the *k*th DM (Boran et al. 2009):
  20 $${\lambda }_{k}=\frac{({\mu }_{k}+{\pi }_{k}\left(\frac{{\mu }_{k}}{{\mu }_{k}+{\nu }_{k}}\right))}{\sum_{k=1}^{l}({\mu }_{k}+{\pi }_{k}\left(\frac{{\mu }_{k}}{{\mu }_{k}+{\nu }_{k}}\right))}\mathrm{and }\sum_{k=1}^{l}{\lambda }_{k}=1$$
- Step 2: Make the combined IF decision matrix based on the DMs’ assessments. To create a combined IF decision matrix in a group decision-making process, all individual decision views must be integrated into a group opinion. For group opinion, the IF weighted average (IFWA) operator suggested by Xu (2007) is employed. The opinion of each decision maker is evaluated by performing the operations in Eq. (21).
  21 $$\begin{array}{c}{r}_{ij}={IFWA}_{\lambda }\left({r}_{ij}^{\left(1\right)},{r}_{ij}^{\left(2\right)},\dots \dots .{r}_{ij}^{\left(l\right)}\right)={r}_{ij}^{\left(1\right)}{\lambda }_{1}\oplus {r}_{ij}^{\left(2\right)}{\lambda }_{2}\oplus \dots \dots \oplus {r}_{ij}^{\left(l\right)}{\lambda }_{l}\\ =\left[1-\prod_{k=1}^{l}{\left(1-{\mu }_{ij}^{\left(k\right)}\right)}^{{\lambda }_{k}},\prod_{k=1}^{l}{\left({\nu }_{ij}^{\left(k\right)}\right)}^{{\lambda }_{k}},\prod_{k=1}^{l}{\left(1-{\mu }_{ij}^{\left(k\right)}\right)}^{{\lambda }_{k}}-\prod_{k=1}^{l}{\left({\nu }_{ij}^{\left(k\right)}\right)}^{{\lambda }_{k}}\right]\end{array}$$
  Here $${r}_{ij}=\left({\mu }_{Ai}\left({x}_{j}\right),{\nu }_{Ai}\left({x}_{j}\right),{\pi }_{Ai}\left({x}_{j}\right)\right)\left(i=\mathrm{1,2}\dots .m;j=\mathrm{1,2}\dots .n\right)$$
- Step 3: Calculate the average solution values (AV).
  The average solution values are calculated with the help of the IF weighted arithmetic mean IWAM operator (Tikhonenko-Kędziak and Kurkowski 2016). The average solution value is obtained by performing the operations in Eq. (22).
  22 $${{\text{AV}}}_{J}={\text{IWAM}}({x}_{ij})=\left[1-\prod_{k=1}^{l}{\left(1-{\mu }_{ij}\right)}^{1/l},\prod_{k=1}^{l}{\left({\nu }_{ij}\right)}^{1/l}\right]$$
- Step 4: Calculation of positive distance from the mean solution (PDA) and negative distance from the mean solution (NDA). The score function in Eq. (17), given in Section 3.2, calculates the PDA and NDA values. This computation is made using the equation that follows.
  23 $${{\text{PDA}}}_{\dot{{\text{I}}}J}={\left[{{\text{PDA}}}_{\dot{{\text{I}}}J}\right]}_{mxn}\frac{{\text{max}}(0,(S\left({x}_{ij}\right)-S\left({AV}_{j}\right)))}{S{(AV}_{j})}$$
  24 $${{\text{NDA}}}_{\dot{{\text{I}}}J}={\left[{{\text{NDA}}}_{\dot{{\text{I}}}J}\right]}_{mxn}=\frac{{\text{max}}(0,\left(S\left({AV}_{j}\right)-S\left({x}_{ij}\right)\right))}{S{(AV}_{j})}$$
- Step 5: Establish the criteria’s weights. It is possible that certain criteria are more important than others. The opinions of each DM on the weighting of each criterion must be combined to get W.
  Let ${W}_{j}^{(k)}=\left[{\mu }_{j}^{\left(k\right)},{\nu }_{j}^{\left(k\right)},{\pi }_{j}^{\left(k\right)}\right]$ be an IF number assigned by the kth DM to criterion Xj. The criterion weights are then calculated using the IFWA operator (Xu 2007):
  25 $$\begin{array}{c}{W}_{j}={{\text{IFWA}}}_{\lambda }\left({W}_{j}^{\left(1\right)},{W}_{j}^{\left(2\right)},\dots \dots .{W}_{j}^{\left(l\right)}\right)={\lambda }_{1}{W}_{j}^{\left(1\right)}\oplus {\lambda }_{2}{W}_{j}^{\left(2\right)}\oplus \dots \dots \oplus {\lambda }_{l}{W}_{j}^{\left(l\right)}\\ =\left[1-\prod_{k=1}^{l}{\left(1-{\mu }_{j}^{\left(k\right)}\right)}^{{\lambda }_{k}},\prod_{k=1}^{l}{\left({\nu }_{j}^{\left(k\right)}\right)}^{{\lambda }_{k}},\prod_{k=1}^{l}{\left(1-{\mu }_{j}^{\left(k\right)}\right)}^{{\lambda }_{k}}-\prod_{k=1}^{l}{\left({\nu }_{j}^{\left(k\right)}\right)}^{{\lambda }_{k}}\right]\\ W=\left[{W}_{1},{W}_{2},{W}_{3}\dots ..{W}_{j}\right]{\text{here}} {W}_{j}=\left({\mu }_{j},{\nu }_{j},{\pi }_{j}\right)\left(j=\mathrm{1,2}\dots .n\right)\end{array}$$
- Step 6: The weighted total positive distance from the average solution and the negative distance from the average solution ($${SP}_{i},{SN}_{i})$$ of the alternatives are calculated using Eqs. (6–7) described in Section 3.1. The normalization of the weighted sum values of the alternatives is performed using Eqs. (8–9) described in Section 3.1.
- Step 7: Calculating the assessment score (AS) and determining the ranking. As a final step, the assessment scores of all alternatives are calculated using Eq. (10) given in Section 3.1. The order of the options is determined by sorting the AS values from largest to smallest. The larger value is determined as the best alternative.

## Application

During the evaluation, a decision group of five academics with extensive experience in the field of energy is constituted. Seven renewable energy sources have been selected for evaluation by the decision group. Renewable energy sources are determined as Wind (Wi), Solar (So), Geothermal (Ge), Biomass (Bi), Wave (W), Hydraulic (Hy), and Hydrogen (Hd). Following that, criteria for evaluating renewable energy sources are chosen from the literature. Economic, technical, socio-political, and environmental factors are commonly used to evaluate renewable energy sources. Unlike other studies, this one includes production and end-use criteria in addition to the commonly used criteria. As a result, the study’s six main criteria and 17 sub-criteria are determined. The explanations regarding the criteria used in the study are as follows.

### Economic criterion (Ec)

This criterion consists of two sub-criteria: (1) Levelized electricity generation cost ($/MWh) and (2) net energy import dependency. (1) *Levelized electricity generation cost* (*$/MWh*) (*Ec1*): It is the average net present cost of electricity generation over the lifetime of a generation facility. Aryanpur et al. (2019), Evans et al. (2009), Klein and Whalley (2015), Ren et al. (2015), Saraswat and Digalwar (2021), Torul Yürek et al. (2021), and Yilan et al. (2020) used this criterion. (2) *Net energy import dependency* (*Ec2*): The net amount of energy an economy must import to reduce fossil fuel consumption and greenhouse gas emissions. Ribeiro et al. (2013) and IEA (2015) use this criterion.

### Technical criterion (T)

This criterion consists of three sub-criteria: efficiency, natural reserve potential, and technological maturity. (1) *Efficiency* (*Te1*): It shows how efficiently energy can be obtained from renewable energy sources. Abdullah and Najib (2016), Ahmad and Tahar (2014), al Garni et al. (2016), Amer and Daim (2011), Arce et al. (2015), Azhar and Ullah (2020), Çolak and Kaya (2017), Ilbahar et al. (2020), Kaya and Kahraman (2010), Mateo (2012), Maxim (2014), Stein (2013), Wang et al. (2009), Wang et al. (2020), Wu et al. (2018), Yilan et al. (2020), and Yuan et al. (2018) used this criterion. (2) *Natural reserve potential* (*Te2*): It is the degree to which a country’s natural climate, natural capital, and technical progress support its production structure. Torul Yürek et al. (2021) used this criterion.

(3) *Technology Maturity (Te3):* It shows how widespread the technology used in renewable energy production is at the regional, national, and international levels. Ahmad and Tahar (2014), al Garni et al. (2016), Amer and Daim (2011), Arce et al. (2015), Beccali et al. (2003), Büyüközkan and Güleryüz (2014), Çolak and Kaya (2017), Ilbahar et al. (2020), Kahraman et al. (2009), Kaya and Kahraman (2010), Mateo (2012), Nigim et al. (2004), Özkale et al. (2017), Ren et al. (2015), Seker and Kahraman (2021), Solangi et al. (2020), Troldborg et al. (2014), Wang et al. (2009), and Wu et al. (2018) used this criterion.

### Socio-political criterion (S)

This criterion consists of three sub-criteria: the reaction of local and non-governmental organizations, job creation, and public policy and financial support. (1) *The reaction of local non-governmental organizations* (*Sp1*): It is the general view of local and non-governmental organizations' views on renewable energy systems. Özkale et al. (2017) used this criterion. (2) *Job creation / Welfare improvement* (*Sp2*): It refers to the employment creation potential of renewable energy technology. Abdullah and Najib (2016), Ahmad and Tahar (2014), al Garni et al. (2016), Amer and Daim (2011), Aryanpur et al. (2019), Beccali et al. (2003), Brand and Missaoui (2014), Çolak and Kaya (2017), Georgopoulou et al. (1997), Ilbahar et al. (2020), Kahraman et al. (2009), Kahraman and Kaya (2010), Kaya and Kahraman (2010), Mateo (2012), Nigim et al. (2004), Ribeiro et al. (2013), Sadeghi et al. (2012), Şengül et al. (2015), Stein (2013), Tasri and Susilawati (2014), Wang et al. (2009), Wu et al. (2018), Yilan et al. (2020), and Yuan et al. (2018) used this criterion. (3) Public policy and financial support (Sp3): Government policies regarding renewable energy technologies include national/international funding sources and government economic support (tariff guarantees, production and investment tax credits, difference contracts, net metering plans, etc.). Büyüközkan and Güleryüz (2016) used this criterion.

### Environmental criterion (En)

This criterion consists of two sub-criteria: influence on the local environment and land requirement. (1) *Influence on the local environment* (*En1*): Noise, odor, emission, waste, natural disaster (fire, explosion), damage to living things (human, animal, plant, etc.), destruction of usable areas, public reaction, is the criterion that expresses its effect on climate change. Abdullah and Najib (2016), Ahmad and Tahar (2014), al Garni et al. (2016), Amer and Daim (2011), Choudhary and Shankar (2012), Daniel et al. (2010), Diakoulaki and Karangelis (2007), Goletsis et al. (2003), Mateo (2012), Özkale et al. (2017), Sadeghi et al. (2012), Tasri and Susilawati (2014), and Troldborg et al. (2014) used this criterion. (2) *Land requirement* (*En2*): It shows the area of land required to build a renewable power plant. (Alkan and Albayrak 2020; Amer and Daim 2011; Aryanpur et al. 2019; Azhar and Ullah 2020; Beccali et al. 2003; Chatzimouratidis and Pilavachi 2008; Choudhary and Shankar 2012; Çolak and Kaya 2017; Evans et al. 2009; Georgopoulou et al. 1997; Ilbahar et al. 2020; Kahraman et al. 2009; Kaya and Kahraman 2010; Mateo 2012; Nigim et al. 2004; Sadeghi et al. 2012; Shakouri G. and Aliakbarisani 2016; Shen et al. 2010; Štreimikiene et al. 2016; Tasri and Susilawati 2014; Troldborg et al. 2014; Wang et al. 2009; Wu et al. 2018).

### Production criterion (Pr)

This criterion consists of two sub-criteria: The reserves-to-production ratio and the Resources-to-production ratio. (1) *Reserves-to-production ratio* (*Pr1*): It is an estimate of the number of years a natural resource field will continue to be productive based on current production rates. (2) *Resources-to-production ratio* (*Pr2*): It is the ratio of the actual energy output of a power plant to the potential output in a given period. These criteria are included in the IEA (2015).

### End-use criterion (Eu)

This criterion consists of five sub-criteria: Industrial, Agricultural, Service/commercial, Household, and Transport energy intensities. (1) *Industrial energy intensities* (*Eu1*): It is the ratio of industry energy consumption to gross industry value-added. (2) *Agricultural energy intensities* (*Eu2*): It is the ratio of agricultural energy consumption to agricultural gross value added. (3) *Service*/*commercial energy intensities* (*Eu3*): It is the ratio of energy consumption in the service sector to the gross value added of the service sector. (4) *Household energy intensities* (*Eu4*): It is the ratio of household energy consumption to population. (5) *Transport energy intensities* (*Eu5*): It is the ratio of energy consumption in the transport sector to the gross value added of the transport sector. These criteria are in the IEA (2015).

The EDAS method, which was recently proposed in the literature and has been effectively applied to many different decision problems, is utilized in evaluating renewable energy sources. It is been suggested as an IF-EDAS approach since the EDAS technique is combined with IFN, which allows for linguistic evaluations in the DMP and gives the DM flexibility to cope with the inherent ambiguity in the DMP. It is required to ascertain the weights of each DM before advancing to the steps of the IF-EDAS approach. The importance degree of five decision-making academicians is determined by their years of experience and publications in this subject. The weights of each of them are calculated considering the operations of Eq. (20). Table 2 summarizes the findings. The academicians' weights are a measure of how much each academician’s opinion will be represented in the combined decision matrix when calculating the combined decision matrix (Appendix 1 contains the IF numbers and values used in Table 2).

**Table 2:** Academic group’s importance ratings

EG	Importance ratings	IF numbers	Λ
Ac1	VI	0.80–0.10	0.243
Ac2	VI	0.80–0.10	0.243
Ac3	I	0.50–0.30	0.188
Ac4	I	0.50–0.30	0.188
Ac5	M	0.50–0.50	0.137

Using the linguistic terms in Table 3, the academic group is asked to evaluate seven renewable energy sources based on 17 criteria. Appendix 2 contains the evaluation results.

**Table 3:** Linguistic terms and IF numbers

Linguistic terms	Abbreviation	Linguistic terms	Abbreviation	IF numbers
Absolutely important	AI	Absolutely good	AG	0.90–0.10
Very important	VI	Very good	VG	0.80–0.05
Important	I	Good	G	0.65–0.25
Medium	M	Medium	M	0.50–0.50
Unimportant	U	Bad	B	0.35–0.55
Very unimportant	VU	Very bad	VB	0.20–0.05
Absolutely unimportant	AU	Absolutely bad	AB	0.10–0.90

The outcomes of the assessments are integrated as group thinking to avoid knowledge loss in expert evaluations, and a combined decision matrix is obtained. The IFWA operator (Xu 2007) is utilized to obtain groupthink. As a result, DMs with various levels of expertise and knowledge are participating equally in the process (Aloini et al. 2018). The IFWA operator is used to combine the linguistic evaluations of five academic teams, resulting in the combined decision matrix shown in Table 4.

**Table 4:** The combined decision matrix

	Ec1			Ec2			Te1		
Wi	0.822	0.106	0.150	0.849	0.125	0.122	0.852	0.123	0.143
So	0.738	0.157	0.145	0.840	0.148	0.137	0.775	0.159	0.137
Ge	0.682	0.157	0.028	0.772	0.175	0.051	0.711	0.250	0.000
Bi	0.639	0.302	0.122	0.730	0.243	0.158	0.691	0.285	0.157
Wa	0.446	0.460	0.143	0.686	0.266	0.143	0.665	0.300	0.157
Hy	0.799	0.084	0.158	0.846	0.109	0.122	0.761	0.141	0.158
Hd	0.564	0.294	0.143	0.711	0.263	0.097	0.756	0.204	0.163
	Te2			Te3			Sp1		
Wi	0.866	0.075	0.182	0.812	0.080	0.141	0.679	0.261	0.102
So	0.882	0.084	0.137	0.701	0.138	0.157	0.647	0.237	0.122
Ge	0.760	0.144	0.051	0.705	0.182	0.163	0.587	0.284	0.000
Bi	0.743	0.151	0.178	0.580	0.291	0.157	0.550	0.377	0.113
Wa	0.746	0.100	0.125	0.534	0.307	0.122	0.533	0.466	0.133
Hy	0.840	0.078	0.178	0.783	0.098	0.157	0.416	0.530	0.125
Hd	0.800	0.091	0.128	0.564	0.294	0.097	0.598	0.356	0.143
	Sp2			Sp3			En1		
Wi	0.752	0.157	0.170	0.818	0.100	0.110	0.633	0.338	0.000
So	0.685	0.172	0.146	0.818	0.100	0.210	0.622	0.270	0.104
Ge	0.687	0.231	0.086	0.787	0.138	0.096	0.607	0.271	0.161
Bi	0.643	0.203	0.128	0.730	0.167	0.146	0.487	0.440	0.125
Wa	0.466	0.454	0.133	0.541	0.320	0.157	0.387	0.488	0.150
Hy	0.657	0.204	0.125	0.701	0.138	0.158	0.676	0.177	0.150
Hd	0.499	0.493	0.125	0.594	0.273	0.146	0.550	0.379	0.159
	En2			Pr1			Pr2		
Wi	0.663	0.286	0.138	0.654	0.228	0.028	0.593	0.283	0.125
So	0.654	0.228	0.120	0.761	0.160	0.087	0.650	0.250	0.104
Ge	0.607	0.271	0.087	0.541	0.365	0.028	0.503	0.358	0.086
Bi	0.519	0.430	0.160	0.519	0.430	0.113	0.636	0.232	0.125
Wa	0.387	0.488	0.150	0.496	0.428	0.128	0.496	0.428	0.159
Hy	0.703	0.150	0.150	0.714	0.129	0.125	0.691	0.150	0.158
Hd	0.549	0.371	0.146	0.599	0.320	0.150	0.524	0.338	0.157
	Eu1			Eu2			Eu3		
Wi	0.676	0.200	0.125	0.676	0.200	0.150	0.479	0.448	0.114
So	0.676	0.200	0.078	0.708	0.148	0.145	0.545	0.345	0.170
Ge	0.633	0.232	0.036	0.633	0.232	0.000	0.545	0.368	0.096
Bi	0.541	0.365	0.125	0.519	0.430	0.102	0.588	0.353	0.172
Wa	0.496	0.428	0.163	0.496	0.428	0.133	0.462	0.444	0.206
Hy	0.691	0.150	0.143	0.714	0.129	0.128	0.539	0.295	0.028
Hd	0.524	0.338	0.133	0.599	0.320	0.133	0.599	0.320	0.163
	Eu4			Eu5					
Wi	0.817	0.125	0.163	0.509	0.446	0.163			
So	0.891	0.090	0.078	0.615	0.273	0.078			
Ge	0.760	0.232	0.086	0.678	0.264	0.086			
Bi	0.776	0.204	0.128	0.679	0.261	0.128			
Wa	0.730	0.177	0.143	0.740	0.142	0.143			
Hy	0.541	0.419	0.128	0.377	0.524	0.128			
Hd	0.789	0.103	0.128	0.740	0.142	0.128			

Each criterion is evaluated by the academic team using the linguistic terms listed in Table 3. Appendix 3 contains the evaluation results.

The importance of the criteria varies depending on the DM, so the weight of each criterion is not equal. To calculate the weight values of the criteria, the IFWA operator and the calculations in Eq. (21) are used. The criterion weights are converted to real numbers using the score function after they have been calculated. The results are shown in Table 5.

**Table 5:** Weight values of criteria

Criteria	Obtained values			S(W)
Ec1	0.829	0.148	0.023	0.841
Ec2	0.798	0.141	0.061	0.829
Te1	0.832	0.092	0.076	0.870
Te2	0.774	0.068	0.158	0.853
Te3	0.847	0.065	0.088	0.891
Sp1	0.525	0.427	0.048	0.549
Sp2	0.784	0.085	0.131	0.850
Sp3	0.852	0.067	0.081	0.893
En1	0.712	0.137	0.151	0.788
En2	0.661	0.275	0.064	0.693
Pr1	0.777	0.114	0.109	0.832
Pr2	0.779	0.148	0.073	0.816
Eu1	0.757	0.135	0.108	0.811
Eu2	0.757	0.135	0.108	0.811
Eu3	0.779	0.148	0.073	0.816
Eu4	0.825	0.157	0.018	0.834
Eu5	0.633	0.233	0.134	0.700

The average solution values are calculated using the IF weighted arithmetic mean (IWAM) operator (Tikhonenko-Kędziak and Kurkowski 2016), and the operations in Eq. (22) are performed. Table 6 displays the calculated average solution values converted to exact numbers by the scoring function.

**Table 6:** The average solution values

	Ec1	Ec2	Te1	Te2	Te3	Sp1	Sp2	Sp3	En1
S(AV)	0.752	0.804	0.777	0.858	0.756	0.618	0.696	0.785	0.628
	En2	Pr1	Pr2	Eu1	Eu2	Eu3	Eu4	Eu5	
S(AV)	0.649	0.678	0.658	0.679	0.693	0.588	0.811	0.688	

Table 7 shows the positive (PDA) and negative (NDA) distances from the average solution calculated using Eqs. (21–22).

**Table 7:** PDA and NDA distances

PDA																	
	Ec1		Ec2		Te1		Te2		Te3		Sp1		Sp2		Sp3		En1
Wi	0.141		0.072		0.113		0.044		0.146		0.147		0.146		0.094		0.031
So	0.051		0.052		0.040		0.048		0.034		0.141		0.087		0.094		0.076
Ge	0.014		0.000		0.000		0.000		0.000		0.054		0.046		0.050		0.064
Bi	0.000		0.000		0.000		0.000		0.000		0.000		0.034		0.000		0.000
Wa	0.000		0.000		0.000		0.000		0.000		0.000		0.000		0.000		0.000
Hy	0.140		0.080		0.042		0.027		0.114		0.000		0.044		0.000		0.193
Hd	0.000		0.000		0.000		0.000		0.000		0.005		0.000		0.000		0.000
PDA																	
	En2		Pr1		Pr2		Eu1		Eu2		Eu3		Eu4		Eu5		
Wi	0.061		0.052		0.000		0.087		0.065		0.000		0.043		0.000		
So	0.099		0.181		0.064		0.087		0.126		0.020		0.110		0.000		
Ge	0.029		0.000		0.000		0.032		0.011		0.001		0.000		0.028		
Bi	0.000		0.000		0.067		0.000		0.000		0.050		0.000		0.031		
Wa	0.000		0.000		0.000		0.000		0.000		0.000		0.000		0.161		
Hy	0.196		0.169		0.171		0.135		0.144		0.058		0.000		0.000		
Hd	0.000		0.000		0.000		0.000		0.000		0.088		0.039		0.161		
NDA																	
	Ec1	Ec2		Te1		Te2		Te3		Sp1		Sp2		Sp3		En1	
Wi	0.000	0.000		0.000		0.000		0.000		0.000		0.000		0.000		0.000	
So	0.000	0.000		0.000		0.000		0.000		0.000		0.000		0.000		0.000	
Ge	0.000	0.007		0.060		0.058		0.000		0.000		0.000		0.000		0.000	
Bi	0.111	0.075		0.095		0.072		0.147		0.051		0.000		0.004		0.166	
Wa	0.344	0.117		0.122		0.041		0.188		0.137		0.273		0.222		0.284	
Hy	0.000	0.000		0.000		0.000		0.000		0.283		0.000		0.004		0.000	
Hd	0.156	0.100		0.001		0.004		0.160		0.000		0.277		0.159		0.068	
NDA																	
	En2	Pr1		Pr2		Eu1		Eu2		Eu3		Eu4		Eu5			
Wi	0.000	0.000		0.005		0.000		0.000		0.123		0.000		0.227			
So	0.000	0.000		0.000		0.000		0.000		0.000		0.000		0.025			
Ge	0.000	0.133		0.130		0.000		0.000		0.000		0.058		0.000			
Bi	0.161	0.197		0.000		0.134		0.214		0.000		0.031		0.000			
Wa	0.307	0.212		0.188		0.214		0.229		0.134		0.043		0.000			
Hy	0.000	0.000		0.000		0.000		0.000		0.000		0.308		0.380			
Hd	0.092	0.057		0.099		0.127		0.077		0.000		0.000		0.000			

The ${SP}_{i}$ and ${SN}_{i}$ values are calculated using Eqs. (6–7), and the IFWA operator criteria weights are combined with the PDA and NDA calculated in the previous phase. ${AS}_{i}$ is calculated by averaging the ${NSP}_{i}\mathrm{ and }{NSN}_{i}$ values, which were calculated by dividing the ${SP}_{i}$ and ${SN}_{i}$ values by their highest values following Eqs. (8–9). Regarding evaluation scores, renewable energy resources are ranked from highest to lowest. The $NS,SNP,SN,NSN,$ and assessment scores obtained during the analysis are shown in Table 8.

**Table 8:** $NS,SNP,SN,NSN$, and rank

		${SP}_{i}$	${NSP}_{i}$	${SN}_{i}$	${NSN}_{i}$	${AS}_{i}$	Rank
Wi	Wind	1.001	0.817	0.264	0.893	0.855	3
So	Solar	1.039	0.848	0.017	0.993	0.921	1
Ge	Geotermal	0.250	0.204	0.372	0.849	0.527	4
Bi	Biomass	0.146	0.119	1.178	0.523	0.321	6
Wa	Wave	0.113	0.092	2.473	0.001	0.046	7
Hy	Hydraulic	1.225	1.000	0.683	0.724	0.862	2
Hd	Hydrojen	0.220	0.180	1.148	0.535	0.357	5

Table 8 shows that solar was the best renewable energy source, with hydraulic coming in second and wind coming in third. Wave is received the lowest score among renewable energy sources.

### Sensitivity analysis

**Fig. 1:**
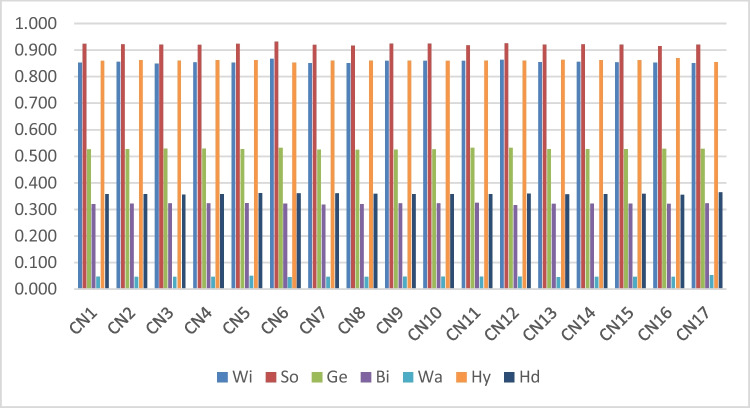
Performance sensitivity analysis at different weights

The criteria weights are crucial in the final step of the decision-making process, which involves ranking the alternatives. The impact of any changes in relative weights on the final ranking should therefore be examined. Because these weights are usually based on experts’ subjective opinions, they impact the ranking of the alternatives. Scenarios that take into account the relative weight of the criteria and depict the issue from various perspectives should be looked at for this aim. In this scenario, ranking alternatives and prioritizing changes in outcomes should be observed, with increasing or decreasing weights for each criterion (Büyüközkan and Güleryüz, 2016). A sensitivity analysis is carried out for this study’s purposes. The other criteria’s weights are left unchanged while the weight of one of the criteria changed. Table 9 displays linguistic terms that have been adjusted for each criterion. The decision model is recalculated for 17 different instances based on the information in this table. The new values that were calculated as a result are presented in Table 10. When the collected findings are evaluated, it became clear that the aforementioned alterations in the criteria had no impact on the options. The ranking remained the same, as shown in Table 9 and 10, and Fig. 1.

**Table 9:** Sensitivity analysis

Case number		Linguistic terms	Rank
CN1	Ec1	I-I-I-I-VI	So˃Hy˃Wi˃Ge˃Hd˃Bi˃Wa
CN2	Ec2	AI-AI-VI-I-I	So˃Hy˃Wi˃Ge˃Hd˃Bi˃Wa
CN3	Te1	M-AI-AI-AI-I	So˃Hy˃Wi˃Ge˃Hd˃Bi˃Wa
CN4	Te2	I-I-I-M-VI	So˃Hy˃Wi˃Ge˃Hd˃Bi˃Wa
CN5	Te3	M-AI-M-VI-AI	So˃Hy˃Wi˃Ge˃Hd˃Bi˃Wa
CN6	Sp1	I-I-I-I-VI	So˃Hy˃Wi˃Ge˃Hd˃Bi˃Wa
CN7	Sp2	M-VI-U-AI-I	So˃Hy˃Wi˃Ge˃Hd˃Bi˃Wa
CN8	Sp3	I-VI-AI-M-İ	So˃Hy˃Wi˃Ge˃Hd˃Bi˃Wa
CN9	En1	I-AI-M-M-M	So˃Hy˃Wi˃Ge˃Hd˃Bi˃Wa
CN10	En2	M-M-AU-M-M	So˃Hy˃Wi˃Ge˃Hd˃Bi˃Wa
CN11	Pr1	I-I-I-I-M	So˃Hy˃Wi˃Ge˃Hd˃Bi˃Wa
CN12	Pr2	M-AI-I-M-U	So˃Hy˃Wi˃Ge˃Hd˃Bi˃Wa
CN13	Eu1	AI-VI-AI-VI-M	So˃Hy˃Wi˃Ge˃Hd˃Bi˃Wa
CN14	Eu2	AI-AI-U-I-M	So˃Hy˃Wi˃Ge˃Hd˃Bi˃Wa
CN15	Eu3	I-AI-AI–AI-I	So˃Hy˃Wi˃Ge˃Hd˃Bi˃Wa
CN16	Eu4	I-M-I-I-VI	So˃Hy˃Wi˃Ge˃Hd˃Bi˃Wa
CN17	Eu5	M-I-VI-VI-AI	So˃Hy˃Wi˃Ge˃Hd˃Bi˃Wa

**Table 10:** Results of sensitivity analysis

	**CN1**	**CN2**	**CN3**	**CN4**	**CN5**	**CN6**	**CN7**	**CN8**	**CN9**
Wi	0.853	0.855	0.849	0.854	0.853	0.867	0.851	0.851	0.859
So	0.923	0.921	0.920	0.919	0.923	0.932	0.919	0.917	0.924
Ge	0.526	0.527	0.529	0.529	0.528	0.532	0.525	0.524	0.525
Bi	0.320	0.322	0.323	0.323	0.324	0.322	0.318	0.319	0.323
Wa	0.047	0.046	0.046	0.046	0.049	0.046	0.046	0.046	0.047
Hy	0.859	0.862	0.861	0.862	0.862	0.853	0.861	0.861	0.861
Hd	0.358	0.358	0.356	0.357	0.361	0.360	0.361	0.359	0.358
	**CN10**	**CN11**	**CN12**	**CN13**	**CN14**	**CN15**	**CN16**	**CN17**	
Wi	0.860	0.859	0.863	0.855	0.856	0.854	0.853	0.851	
So	0.924	0.918	0.925	0.920	0.921	0.920	0.915	0.920	
Ge	0.526	0.532	0.532	0.527	0.527	0.527	0.528	0.528	
Bi	0.323	0.325	0.316	0.321	0.322	0.322	0.322	0.323	
Wa	0.047	0.047	0.047	0.046	0.046	0.046	0.046	0.053	
Hy	0.860	0.860	0.861	0.863	0.862	0.862	0.870	0.854	
Hd	0.358	0.358	0.360	0.357	0.358	0.359	0.355	0.364

## Conclusions

The amount and type of energy used in a country are important for fundamental indicators such as economic growth, sustainable growth, environmental quality, quality of life, and social development. The intensive use of fossil energy resources causes environmental pollution, adversely affects public health, increases energy dependency, and increases the foreign trade deficit for the countries that import these resources. One of the most important ways to reduce these adverse effects of fossil energy sources is the widespread use of alternative renewable energy sources such as solar, wind, geothermal, biomass, wave, hydraulic, and hydrogen.

Turkey is a country that imports most of its fossil-based energy consumption and is highly dependent on foreign sources. Turkey’s national energy production cannot meet the demand for energy supply, and this situation causes the gap between supply and demand to increase continuously. However, the savings gap and the high cost of foreign borrowing in Turkey necessitate investment choices in the most productive areas. On the other hand, although Turkey has a high potential in renewable energy production, it is necessary to determine policies and targets and make serious investments in this field to bring renewable energy production to a certain level. In this context, it is an important question that needs to be answered about which renewable energy type to choose while making an investment choice and which criteria to consider when making this choice.

This study ranked renewable energy alternatives in Turkey using the IF-EDAS method by having five academicians and experts, who are decision-makers in the energy field and who evaluate 7 renewable energy options with 17 criteria. The number of studies conducting the IF-EDAS method is very few in the literature. By following this method, this study contributes to the literature on renewable energy source selection, and IF sets literature. In addition, the study used two main and seven sub-criteria, which were not used in the literature before, to evaluate alternative renewable energy sources with IF-EDAS analysis. Hence, this paper employs those criteria in the relevant literature for the first time. The study’s main findings are that solar energy is the best renewable energy source, hydraulic energy is the second best renewable energy source, and wind is the third best renewable source for Turkey. Wave is received the lowest score among renewable energy sources. Another important finding is that in the basic “criteria evaluation” for Turkey’s renewable energy source, the first criterion is “Technology Maturity,” the second criterion is “Efficiency,” and the third criterion is “Levelized electricity generation cost.” The criterion with the lowest degree is “The reaction of local, non-governmental organizations.”

The priority ranking for renewable energy production and the criteria ranking for renewable energy has important implications for policy recommendations. First of all, while wind and hydro-energy are generally recommended for Turkey in the empirical literature, and their share in total renewable energy production is relatively high, the application of this study recommends solar energy. Since 2010, the share of hydro in Turkey’s total renewable energy has decreased from 90% to around 60%, and the share of wind has increased from 9 to 18%. Solar energy in Turkey is a renewable energy source that has been invested more in recent years due to its high potential, and its share in renewable energy is around 7% as of 2022. Developments in these rates also confirm that Turkey increasingly prefers solar energy in renewable energy.

The fact that many regions in Turkey have high sunshine hours and that solar energy investment costs and technology are more suitable for Turkey confirms the results of the application. Considering the limited reserves of fossil energy resources and their adverse effects on the environment, the investments in solar energy and other renewable energy resources can be given more priority for Turkey. In addition, in parallel with the findings of the study, providing public support at the regional and national levels for the technology to be used in renewable energy production to reach the required maturity and encouraging foreign direct investments at the international level will be beneficial in terms of both the sustainability of resources and desirable macroeconomic effects. In future studies, the criteria included in this analysis can be varied to ensure optimum decisions in the renewable energy sector. The proposed method in this study can be compared with various fuzzy numbers or expanded and compared with various MCDM methods following an integrated approach.

## Data Availability

The data is openly available on the website of the World Development Indicators (WDI), International Energy Agency (IEA) and the KOF Swiss Economic Institute.

## Appendix 1

See Table 11

**Table 11:** Linguistic terms used to determine the importance levels of the expert team

LT	IFN
VI (very important)	0.80–0.10
I(important)	0.50–0.30
M (medium)	0.50–0.50
U (unimportant)	0.30–0.50
VU (very unimportant)	0.20–0.70

## Appendix 2

See Table 12, 13, 14, 15, and 16

**Table 12:** Ac 1’s opinions

Criteria	Wi	So	Ge	Bi	Wa	Hy	Hd
Ec1	AG	M	G	M	B	G	M
Ec2	AG	G	M	B	B	G	B
Te1	AG	M	G	G	M	M	G
Te2	AG	AG	AG	G	G	AG	AG
Te3	AG	M	M	M	B	G	M
Sp1	G	G	G	G	M	B	G
Sp2	AG	G	M	M	B	M	M
Sp3	AG	AG	AG	AG	VB	M	G
En1	M	M	G	B	VB	M	VB
En2	G	G	G	M	VB	G	B
Pr1	G	AG	M	M	VB	M	B
Pr2	B	G	M	G	VB	M	B
Eu1	G	G	M	M	VB	M	B
Eu2	G	G	M	M	VB	M	B
Eu3	B	B	B	B	VB	B	B
Eu4	AG	AG	AG	AG	B	AB	M
Eu5	M	G	M	M	G	AB	M

**Table 13:** Ac 2’s opinions

Criteria	Wi	So	Ge	Bi	Wa	Hy	Hd
Ec1	VG	VG	VG	G	G	VG	VG
Ec2	AG	AG	AG	AG	AG	AG	AG
Te1	AG	AG	AG	AG	AG	AG	AG
Te2	VG	VG	VG	VG	VG	VG	VG
Te3	VG	VG	VG	VG	VG	VG	VG
Sp1	M	M	M	M	M	M	M
Sp2	G	G	G	G	G	G	G
Sp3	VG	VG	VG	VG	VG	VG	G
En1	G	G	G	G	G	G	G
En2	G	G	G	G	G	G	G
Pr1	G	G	G	G	G	G	G
Pr2	G	G	G	G	G	G	G
Eu1	G	G	G	G	G	G	G
Eu2	G	G	G	G	G	G	G
Eu3	G	G	G	G	G	G	G
Eu4	AG	AG	AG	AG	AG	AG	AG
Eu5	M	M	G	G	M	G	G

**Table 14:** Ac 3’s opinions

Criteria	Wi	So	Ge	Bi	Wa	Hy	Hd
Ec1	AG	AG	B	B	VB	AG	B
Ec2	AG	AG	M	M	G	AG	B
Te1	AG	AG	M	M	G	VG	B
Te2	AG	AG	M	AG	VG	AG	VG
Te3	VG	VG	B	B	VB	AG	B
Sp1	G	G	VB	B	AB	M	VB
Sp2	VG	VG	B	B	VB	VG	VB
Sp3	VG	VG	B	B	VB	VG	VB
En1	M	M	B	B	VB	VG	VB
En2	M	M	B	B	VB	VG	VB
Pr1	G	G	B	B	AB	VG	VB
Pr2	G	G	M	B	AB	VG	VB
Eu1	G	G	M	B	AB	VG	VB
Eu2	G	G	M	B	AB	VG	VB
Eu3	M	G	AB	B	AB	B	VB
Eu4	VG	AG	B	B	AB	B	VG
Eu5	B	M	AG	G	VG	B	VG

**Table 15:** Ac 4’s opinions

Criteria	Wi	So	Ge	Bi	Wa	Hy	Hd
Ec1	G	G	G	G	M	VG	M
Ec2	G	AG	VG	G	G	VG	AG
Te1	M	G	G	M	M	VG	G
Te2	VG	AG	G	M	G	VG	M
Te3	G	G	AG	M	M	VG	M
Sp1	G	G	G	G	M	B	M
Sp2	M	G	AG	VG	M	G	M
Sp3	M	M	G	M	M	G	M
En1	M	G	M	M	B	VG	M
En2	M	G	G	M	B	VG	B
Pr1	M	G	M	M	B	VG	G
Pr2	G	G	M	G	B	VG	G
Eu1	G	G	VG	M	B	VG	G
Eu2	G	VG	VG	M	B	VG	G
Eu3	M	G	G	M	B	VG	G
Eu4	M	AG	M	G	VG	B	VG
Eu5	G	VG	M	G	VG	B	VG

**Table 16:** Ac 5’s opinions

Criteria	Wi	So	Ge	Bi	Wa	Hy	Hd
Ec1	G	G	VG	AG	B	VG	B
Ec2	G	G	AG	AG	M	AG	B
Te1	VG	VG	M	M	B	M	AG
Te2	AG	AG	G	G	VG	G	VG
Te3	VG	G	G	M	M	G	B
Sp1	AG	VG	VG	M	M	VB	AG
Sp2	G	G	VG	VG	M	G	M
Sp3	AG	AG	AG	G	M	G	VG
En1	AG	VG	G	M	B	M	AG
En2	AG	VG	G	M	B	M	AG
Pr1	VG	VG	G	M	M	VG	AG
Pr2	G	G	G	VG	M	G	G
Eu1	VG	VG	G	G	M	G	G
Eu2	VG	VG	G	M	M	VG	B
Eu3	VB	VB	VB	AG	VB	VB	AG
Eu4	G	VG	M	M	G	VB	VG
Eu5	M	M	G	AG	AG	VB	AG

## Appendix 3

See Table 17

**Table 17:** Academic team’s criteria assessments

Criteria	Ac1	Ac2	Ac3	Ac4	Ac5
Ec1	AI	I	AI	I	AI
Ec2	I	I	AI	AI	VI
Te1	AI	VI	AI	VI	M
Te2	VI	VI	VI	VI	M
Te3	VI	VI	AI	AI	VI
Sp1	M	I	M	M	U
Sp2	I	I	VI	M	AI
Sp3	AI	I	VI	AI	VI
En1	VI	I	I	VI	M
En2	I	I	I	I	M
Pr1	I	VI	AI	I	VI
Pr2	M	VI	AI	I	AI
Eu1	M	VI	AI	I	VI
Eu2	M	VI	AI	I	VI
Eu3	M	VI	AI	I	AI
Eu4	M	AI	AI	AI	I
Eu5	I	M	VI	M	I

## Abbreviations

AHP: Analytical hierarchy process
ANP: Analytical network process
CHP: Combined heat and power
COPRAS: Complex proportional assessment
DEMATEL: Decision-making trial and evaluation laboratory
EDAS: Evaluation based on distance from average solution
ELECTRE: Elimination and choice translating reality
FMEA: Failure mode and effect analysis
G-AHP: Grey analytic hierarchy process
GDP: Gross domestic production
GHG: Greenhouse gas
IAEA: International Atomic Energy Agency
IEA: International Energy Agency
IF: Intuitionistic fuzzy
IFWA: Intuitionistic fuzzy weighted averaging
KTOE: Kilotonne of oil equivalent
LNG: Liquefied natural gas
MCDM: Multi-criteria decision making
MENR: Ministry of Energy and Natural Resources (Turkey)
MULTIMOORA: The multi-objective optimization by ratio analysis
PROMETHEE: Preference Ranking Organization Method for Enrichment Evaluations
PV: Photovoltaic
R&D: Research and development
SIMUS: Sequential interactive modeling for urban systems
TOPSIS: Technique for Order Preference by Similarity to Ideal Solution
TPES: Total primary energy supply
VIKOR: VlseKriterijumska Optimizacija I Kompromisno Resenje (Multicriteria Optimization and Compromise Solution)
WASPAS: Weighted aggregated sum product assessment
WSM: Weighted sum method
